# Physiological and Molecular Responses of Six Apple Rootstocks to Osmotic Stress

**DOI:** 10.3390/ijms22158263

**Published:** 2021-07-31

**Authors:** Yasmine S. Hezema, Mukund R. Shukla, Murali M. Ayyanath, Sherif M. Sherif, Praveen K. Saxena

**Affiliations:** 1 Gosling Research Institute for Plant Preservation, Department of Plant Agriculture, University of Guelph, Guelph, ON N1G 2W1, Canada; yhezema@uoguelph.ca (Y.S.H.); mshukla@uoguelph.ca (M.R.S.); ayyanath@uoguelph.ca (M.M.A.); 2 Department of Horticulture, Damanhour University, Damanhour 22713, El-Beheira, Egypt; 3 Alson H. Smith Jr. Agricultural Research and Extension Center, School of Plant and Environmental Sciences, Virginia Tech, Winchester, VA 22602, USA

**Keywords:** apple rootstocks, osmotic stress, relative water content, abscisic acid, water use efficiency, osmotic responsive genes

## Abstract

The growth and productivity of several apple rootstocks have been evaluated in various previous studies. However, limited information is available on their tolerance to osmotic stress. In the present study, the physiological and molecular responses as well as abscisic acid (ABA) levels were assessed in six apple rootstocks (M26, V3, G41, G935, B9 and B118) osmotically stressed with polyethylene glycol (PEG, 30%) application under greenhouse conditions. Our results showed that V3, G41, G935 and B9 had higher relative water content (RWC), and lower electrolyte leakage (EL) under stress conditions compared to M26 and B118. Additionally, water use efficiency (WUE) was higher in V3, G41 and B9 than M26, which might be partially due to the lower transpiration rate in these tolerant rootstocks. V3, G41 and B9 rootstocks also displayed high endogenous ABA levels which was combined with a reduction in stomatal conductance and decreased water loss. At the transcriptional level, genes involved in ABA-dependent and ABA-independent pathways, e.g., *SnRK*, *DREB*, *ERD* and *MYC2*, showed higher expression in V3, G41, G935 and B9 rootstocks compared to M26 in response to stress. In contrast, *WRKY29* was down-regulated in response to stress in the tolerant rootstocks, and its expression was negatively correlated with ABA content and stomatal closure. Overall, the findings of this study showed that B9, V3 and G41 displayed better osmotic stress tolerance followed by G935 then M26 and B118 rootstocks.

## 1. Introduction

Apple is one of the most important cultivated fruit trees all over the world. Climate changes and their potential negative effects on apple crop production and sustainability highlight the urgent need for developing stress-resilient cultivars of apple varieties and rootstocks. The effects of several commercial apple rootstocks on the growth and yield parameters of scion varieties have been intensively investigated in several locations in the US and Canada [1,2,3,4,5], yet only a few reports have focused on the tolerance of apple rootstocks to abiotic stress [6,7,8,9]. In the present study, six apple rootstock genotypes (M26, V3, G41, G935, B9 and B118) were evaluated for their responses to osmotic stress. Geneva rootstocks including G41 and G935, have been developed by the Geneva apple rootstock breeding program and are distributed by several tree fruit nurseries worldwide. G41 has a similar size to M9 and a smaller size than M26, while G935 has vigor similar to M26. The size of the tree may differ depending on the scion’s cultivar [4,5]. Both G41 and G935 are resistant to fire blight and tolerant to crown rot [10] and both appear to be very winter hardy [5,11]. The Budagovsky rootstocks including B9 and B118 were generated in Russia. These rootstocks show a fair degree of cold hardiness, moderate resistance to fire blight, and resistance to crown rot [5,6]. B9 shows less vigor than M26, while B118 is more vigorous than B9 [3]. V3 is one of the Vineland rootstocks series that originated in Canada. V3 is slightly similar to M9, smaller than M26 but more resistant to fire blight than M26 [10,12]. M26 is among Malling rootstocks that were selected at the East Malling Research Station at Kent, England. M26 is known to be very susceptible to fire blight and moderately susceptible to crown rot [10].

The reduction in water potential is the primary symptom of osmotic stress induced by drought, salinity, cold and heat stresses. Under osmotic stress, rootstocks may affect several physiological, biochemical and molecular aspects of the scion including stomatal conductance (g_s_), transpiration rate (E), net photosynthesis (P_N_), abscisic acid (ABA) content, osmolytes accumulation, antioxidant activity, root architecture and transcriptional regulation of the osmotic-related genes (ORGs) [9,13,14]. For instance, both M9 and B9 rootstocks were found to improve the drought tolerance of ‘Ambrosia’ scion by showing better water use efficiency (WUE) and P_N_ than G202, M26, or G935 [15]. Also, ‘Gale Gala’ apple cultivar showed better drought tolerance when grafted to *Malus sieversii* than *Malus hupehensis*, as the former promoted better growth, P_N_, total biomass, chlorophyll content and relative water content (RWC) [7]. The tolerance response caused by *M. sieversii* was also associated with reduction in reactive oxygen species (ROS) accumulation in the scion leaves and the rootstock roots and increase in antioxidant enzyme activity like superoxide dismutase, catalase, ascorbate peroxidase and glutathione reductase [7]. G202, G214, and G935 rootstocks also improved the tolerance of ‘Fuji’ to drought stress than M26 and CG5087 rootstocks through maintaining leaf water potential, showing a better vertical growth rate, higher fine root dry weight and higher root to shoot biomass ratio [8]. Rootstocks G202, G814 and Marubakaido/M9 interstock also improved the tolerance of ‘Maxi Gala’ grafted tree when exposed to short-term waterlogging by producing new adventitious roots [16].

ABA content is elevated in response to osmotic stress elicited by drought, salinity, cold and heat stresses [6,17,18]. The phytohormone ABA is a vital root-produced signal [19] that regulates leaf water status under different abiotic stresses through coordinating stomatal movement [6]. Moreover, the reduction of shoot growth rate, leaf expansion rate, and leaf emergence rate correlates negatively with the initial increase in ABA in apple seedlings under water stress [17]. The reduction of stomatal conductance (g_s_) and transpiration rate (E) in M9, the dwarfing apple rootstock, resulted in better drought tolerance than MM.111, the vigorous rootstock, and the tolerance was associated with higher ABA content in M9 than MM.111 under short-term drought stress [3,6]. ABA also induces the expression of several ORGs that are involved in enhancing plant tolerance to osmotic stress [20]. These genes are mainly involved in plant hormone synthesis, plant hormones signal transduction pathways, osmolytes accumulation, antioxidant activity or root growth control [14,20,21].

The increase in ABA in response to stress activates the transcription of several stress-related genes that have ABA-responsive elements (ABREs) in their promoter regions. Sucrose non-fermenting-1 related protein kinase 2 (SnRK2) is a key regulator of the ABA signaling pathway [22]. The role of SnRK2 in many developmental processes, including seed maturation, dormancy, and germination and stomatal movement during drought periods, has been demonstrated [23,24]. Dehydration responsive element binding (DREB) proteins play a critical role in plant tolerance to several abiotic stresses [25,26,27]. There are 68 *MdDREB* members identified in apple [28], many of them are up-regulated in response to drought, salt, cold and heat stress [28]. Furthermore, the overexpression of *MpDREB2A* enhanced drought tolerance in *Arabidopsis* by promoting root system development [29]. The early responsive to dehydration (ERD) was also shown to be involved in ABA-dependent and ABA-independent signaling transduction pathways in response to abiotic stresses [30,31,32,33]. WRKY is a large regulatory protein family with a vital role in plant abiotic and biotic defense via regulation of the transcriptional level of genes involved in salicylic acid (SA) biosynthesis and signaling pathways [34,35,36]. Overexpression of apple *MdWRKY30* has been proven to improve osmotic and salt stress tolerance in apple callus through transcriptional regulation of stress-responsive genes [35]. MYB is another large family that has been widely studied in several plant species. Many MYB members play important roles in abiotic tolerance in plants through hormone signaling pathways including that of ABA and the regulation of secondary metabolism and the cell cycle [37,38]. Overexpression of apple *MdMYB121* in tomato and apple enhanced the tolerance to salinity, drought, and cold stresses [38,39,40]. MYC2 is another transcription factor that is known to regulate jasmonic acid (JA)-mediated resistance and induce tolerance to pathogen infection [40,41]. MYC2 also acts as a positive regulator of ABA signaling pathway in addition to its role in improving tolerance to drought and osmotic stresses [42,43]. Nonexpressor of pathogenesis-related (NPR1) gene is associated with SA and systemic acquired resistance [44,45]. *NPR1* is up-regulated in apple roots subjected to drought stress [45]. NPR1 enhances plant tolerance to drought stress by decreasing stomatal conductance, oxidative stress and elevating activity levels of antioxidant enzymes [45,46]. The role of the plant proton pumps Vacuole H^+^-ATPASE (*MdVHAs*) in improving apple plants tolerance to PEG and sodium chloride (NaCl)-induced osmotic stresses has also confirmed. Indeed, the overexpression of apple *MdVHAs* in tobacco and tomato improved lateral root growth, showed better stomatal closing, and led to better osmotic adjustment under osmotic stress conditions compared to wild-type plant responses [47,48,49].

Rootstocks perform differently under stress depending on their tolerance [8,14]. Therefore, suitable rootstock selection is a limiting factor that affects orchard’s productivity and economics. We hypothesized that the physiological, biochemical and molecular changes of rootstocks subjected to osmotic stress would be affected by their tolerance potential to osmotic stress. The aim of the present work was to explore the differences among six apple rootstock genotypes (M26, V3, G41, G935, B9 and B118) to determine their possible tolerance to osmotic stress and the relationship between the stress-mediated physiological changes and the ABA levels. The study was complemented at the molecular level with monitoring the expression of some vital ORG genes in the tested rootstocks under osmotic stress.

## 2. Results

### 2.1. Physiological Changes in Response to Osmotic Stress

**Figure 1:**
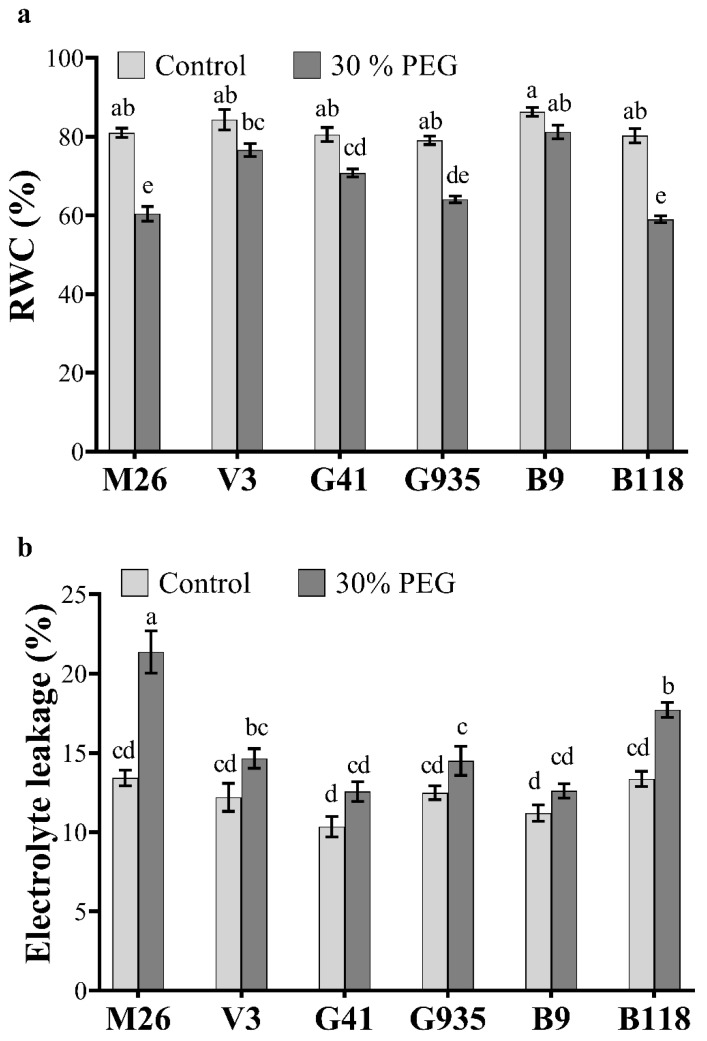
Physiological changes in six apple rootstocks in response to osmotic stress. Relative water content (RWC) (**a**) and electrolyte leakage (EL) (**b**) in six apple rootstocks (M26, V3, G41, G935, B9 and B118) were assessed three days after treatment with 30% PEG. Means from all treatments were compared with each other using Tukey’s test. Vertical bars represent the mean ± SEM of three biological replicates (three plants each). Bars with no common letters are significantly different (*p* < 0.05).

The current study compared the effect of osmotic stress induced by 30% polyethylene glycol (PEG) on M26, V3, G41, G935, B9 and B118 apple rootstocks. When exposed to osmotic stress for 3 consecutive days, the leaf relative water content (RWC) decreased in all tested rootstocks, with a significant reduction of 25%, 12%, 19% and 26% in M26, G41, G935 and B118, respectively, compared to the control (unstressed) plants (Figure 1a). V3 and B9 plants showed insignificant reduction in their water content under stress conditions. Also, no significant differences in RWC were found among different rootstocks under control conditions. RWC was significantly higher in B9, V3 and G41 compared to M26 and B118 under osmotic stress (Figure 1a). The electrolyte leakage (EL) also showed a significant increase of 37% and 24.3% in M26 and B118, respectively, after the PEG treatment as compared to the control (Figure 1b). No significant differences in EL were found among V3, G41, G935 and B9 after stress. Similarly, no differences in EL were observed among all rootstocks under normal conditions (Figure 1b).

**Figure 2:**
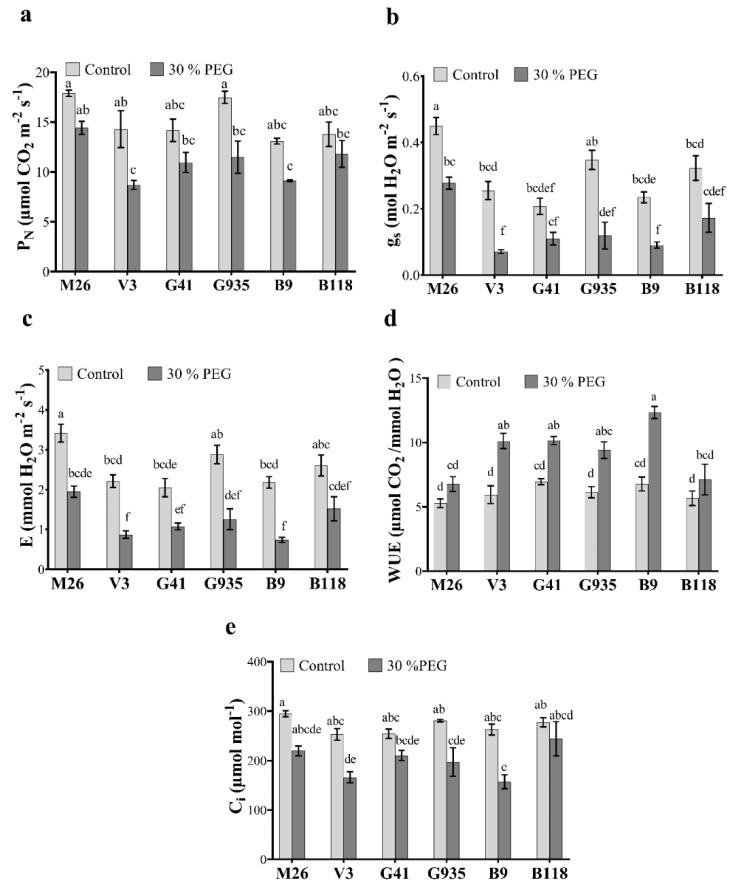
Changes in photosynthesis-related parameters in six apple rootstocks under osmotic stress conditions. Photosynthesis rate (P_N_) (**a**), stomatal conductance (g_s_) (**b**), transpiration rate (E) (**c**), WUE (P_N_/E) (**d**) and intercellular CO_2_ concentration (C_i_) (**e**) observed in control and osmotic-stressed apple rootstocks (M26, V3, G41, G935, B9 and B118) three days after treatment with 30% PEG Means from all the treatments were compared with each other using Tukey’s test. Vertical bars represent the mean ± SEM of three biological replicates (three plants each). Bars with no common letters are significantly different (*p* < 0.05).

The net photosynthetic rates (P_N_), stomatal conductance (g_s_), transpiration rate (E), inner CO_2_ (C_i_) and water use efficiency (WUE) were measured three days after the PEG (30%) treatment. All rootstocks showed a reduction in P_N_ under osmotic stress with a significant decrease of 39% and 34.4% observed in V3 and G935, respectively, compared to V3 and G935 control plants (Figure 2a). V3 and B9 revealed a significant decline in P_N_ compared to M26 under osmotic stress conditions (Figure 2a). Stomatal conductance (g_s_) showed a significant reduction of 38%, 72%, 52 and 61% under osmotic stress in M26, V3, G935 and B9 rootstocks, respectively, and a nonsignificant decrease of 47%, 46% in G41 and B118, respectively, compared to control plants (Figure 2b). Among genotypes, M26 showed the highest g_s_ under control and stress conditions with a nonsignificant difference compared to G935 under control and to B118 under stress conditions (Figure 2b). In a similar fashion, the transpiration rate exhibited a significant decline of 42%, 60%, 56% and 66% in M26, V3, G935 and B9, respectively, and a nonsignificant decline of 47 and 41% in G41 and B118, respectively, compared to rootstocks growing under control conditions (Figure 2c). Among all rootstocks, M26 showed the highest transpiration rate under control and osmotic stress conditions, and was significantly different than V3, G41 and B9 under control conditions and V3 and B9 under osmotic stress (Figure 2c). WUE increased significantly by 41%, 31.5%, 34.8%, and 45% under osmotic stress conditions in V3, G41, G935 and B9, respectively (Figure 2d). The same rootstocks also showed higher WUE compared to M26 under osmotic stress conditions (Figure 2d). The lowest reduction in C_i_ content after osmotic stress treatment was detected by 40% in B9 followed by 34.3% in V3 and 29.8% in G935 compared to the same rootstocks under control conditions (Figure 2e). Although no significant differences in C_i_ content were noticed among the rootstocks under control or osmotic stress conditions except for B118 that had lower C_i_ compared to B9 under stress conditions.

### 2.2. Changes in ABA Levels under Osmotic Stress 

**Figure 3:**
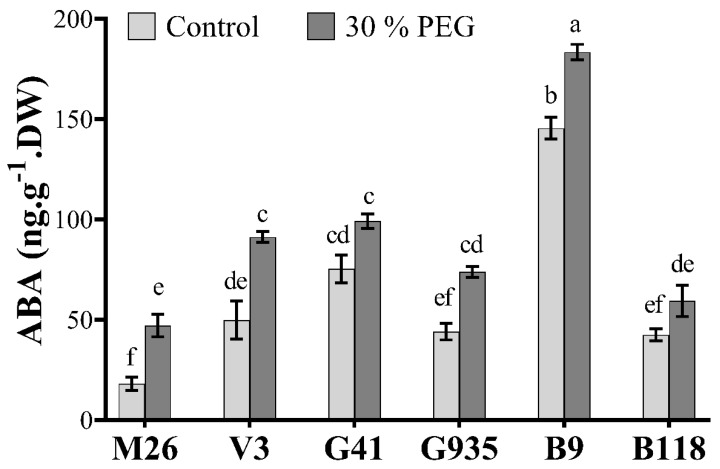
Evaluation of ABA levels in six apple rootstocks (M26, V3, G41, G935, B9 and B118) in control and PEG (30treatment. Means from all the treatments were compared with each other using Tukey’s test. Vertical bars represent the mean ± SEM of three biological replicates (three plants each). Bars with no common letters are significantly different (*p* < 0.05).

The PEG-induced osmotic stress increased ABA levels in all tested rootstocks with a significant increase in M26, V3, G935 and B9 by 61.5%, 45%, 40% and 20.6%, respectively (Figure 3). The concentration of ABA differed among the rootstocks under control and osmotic stress conditions, although it was not always statistically significant. In general, B9 had the highest ABA content under both control and stress conditions. In contrast, M26 had the lowest ABA level under control and stress conditions. Besides, V3, G41, G935 and B9 showed significantly higher ABA levels than M26 under osmotic stress 

### 2.3. Changes in ORGs Gene Expression under Osmotic Stress

**Figure 4:**
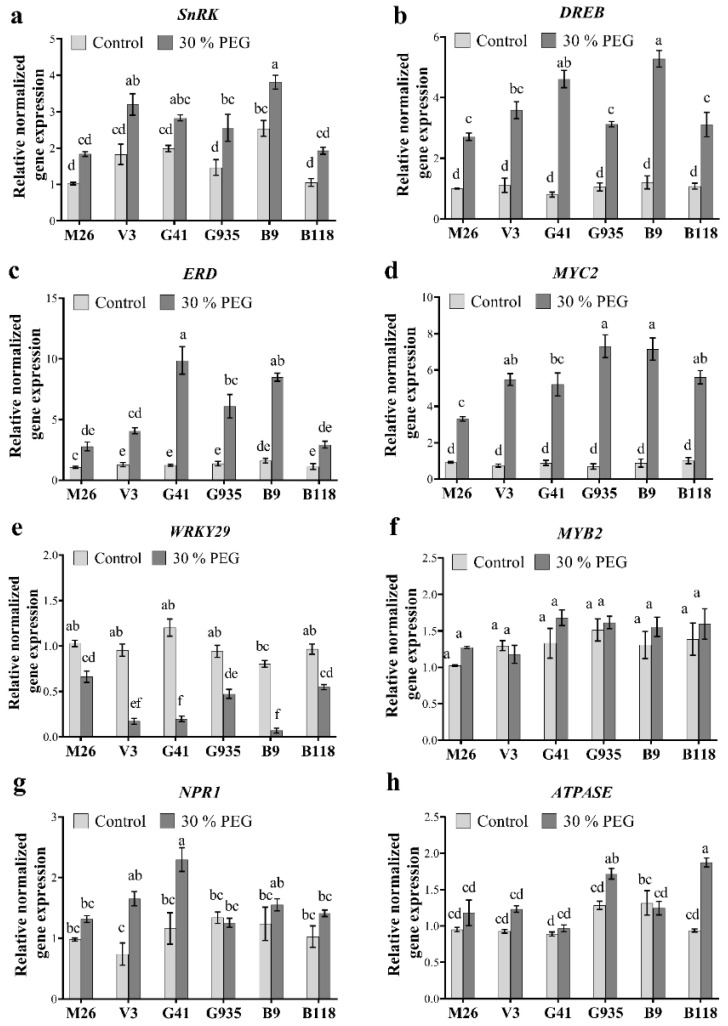
Expression profiles of eight ORGs (**a**–**h**) in six apple rootstocks (M26, V3, G41, G935, B9 and B118) exposed to 30% PEG treatment for 3 days. Genes differentially expressed in plants under stress conditions were normalized with *MdEF1A.* Means from all the treatments were compared with each other using Tukey’s test. Vertical bars represent the mean ± SEM of three biological replicates (three plants each). Bars with no common letters are significantly different (*p* < 0.05).

To evaluate the molecular response to osmotic stress in the selected apple rootstocks, the expression of eight osmotic responsive genes (ORGs) was determined after three days of stress. All the tested ORGs showed significant regulation under osmotic stress except for *MYB2* which remained stable under stress in all tested rootstocks (Figure 4f). The expression of *SnRK* showed significant differences among the six rootstocks under control and osmotic stress conditions. Transcript levels of *SnRK* in B9 were significantly higher compared to M26, G935 and B118 under control and osmotic stress conditions (Figure 4a). However, within the same rootstock, only V3, G935 and B9 showed a significant upregulation in response to osmotic stress. The expression level of *DREB*, *ERD*, *MYC2*, *WRKY29*, *NPR1* and *ATPASE* among rootstocks was statistically similar under control conditions, but significantly different under stress. Osmotic stress induced marked upregulation in the expression level of *DREB* in all rootstocks with the highest upregulation of 5.3 and 4.6-fold change in B9 and G41, respectively (Figure 4b). Similarly, *ERD* showed significant upregulation of 4, 10, 6 and 8.5-fold in V3, G41, G935 and B9, respectively, in response to the PEG treatment (Figure 4c). Transcripts of *MYC2* also increased significantly in all rootstocks after the stress treatment, with the highest upregulation being observed in B9 and G935 followed by V3, G41 and B118 and the lowest upregulation being observed in M26 (Figure 4d). As for *WRKY29* expression, significant down-regulation was detected in all rootstocks after osmotic stress. Moreover, V3, G41 and B9 showed the lowest *WRKY29* expression under osmotic stress, followed by G935 and B118 then M26. A significant increase of 2 and 2.3-fold in gene expression was detected for *NPR1* in V3 and G4 plants, respectively, after the stress treatment (Figure 4g). *ATPASE* expression level also showed significant upregulation in G935 and B118 in response to osmotic stress (Figure 4g).

**Figure 5:**
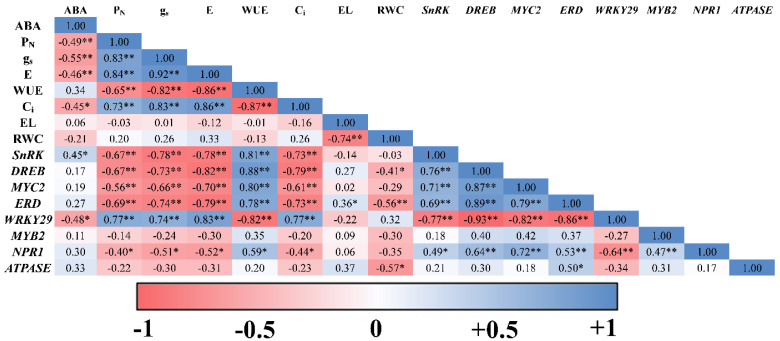
Correlation matrix to investigate the dependence between ABA concentration, physiological parameters (P_N_, g_s_, E, WUE, C_i_, EL and RWC) and transcript levels of ORGs (*SnRK*, *DREB*, *MYC2*, *ERD*, *WRKY29*, *MYB2*, *NPR1* and *ATPASE*) in all apple rootstocks after treatment with 30% PEG. The blue color indicates a positive correlation and the red color indicates a negative correlation. Asterisk(s) indicate(s) significant differences at * *p* < 0.05, ** *p* < 0.01 according to Pearson correlation test.

Strong negative correlations were recorded between ABA on one side and the physiological variables P_N_, g_s_, E and C_i_ on the other side. Meanwhile, ABA showed a positive, yet insignificant, correlation with WUE. Moreover, ABA was positively correlated with *SnRK* and negatively correlated with *WRKY29* (Figure 5). There was also a strong positive correlation between WUE on one side and transcript levels of *SnRK*, *DREB*, *ERD*, *MYC2*, *NPR1* on the other side. *WRKY29* also showed a highly negative correlation with the WUE and a highly positive association with P_N_, g_s_, E and C (Figure 5).

## 3. Discussion

### 3.1. The Physiological Changes under Osmotic Stress in Apple Rootstocks

Understanding the adaptive mechanisms underlying osmotic stress tolerance in apple rootstocks has become increasingly important, especially in the context of climate change where abiotic stresses are expected to exacerbate. In general, plants exposed to osmotic stress undergo several physiological, biochemical and molecular changes to mitigate and diminish stress influences [50]. In the current study, we studied the effect of PEG-induced osmotic stress on six apple rootstocks. Apple rootstocks subjected to osmotic stress for three days showed low RWC and high EL than rootstocks growing under control conditions (Figure 1). These results agree with Kautz et al. (2015) [9] who reported a reduction in RWC in apple plants exposed to PEG and drought stresses. Leaf water potential drives several physiological, biochemical and molecular changes in stressed plants [21,50,51,52]. Therefore, RWC is an important indicator of plant water status and plant tolerance potential. With B9 and V3 showing insignificant change in RWC between PEG-treated and untreated trees, it could thus be concluded that both rootstocks are relatively more tolerant to osmotic stress than the rest of the tested rootstocks, especially M26 and B118. The increase in EL reflects the damage to cellular membranes caused by oxidative stress and consequently the increase in cell permeability [13,21], which again confirms the relative sensitivity of M26 and B118 to osmotic stress. The EL of these two rootstocks significantly increased with exposure to PEG. An earlier study reported that M26 is very susceptible to drought stress [53]. Also, Choi et al. (2020) [8] reported that ‘Fuji’ variety showed better drought tolerance when grafted on G935 than M26.

As an adaptation mechanism to alleviate water loss, plants reduce stomatal conductance (g_s_) which in turn results in a reduction in transpiration rate (E) and intercellular CO_2_ (C_i_) content [8,52,54]. In addition, the lower diffusion of CO_2_ through the leaf causes a decline in photosynthesis in stressed rootstocks compared to control rootstocks [8,9]. Our study showed a significant reduction in all these physiological parameters, except WUE, in the relatively tolerant B9 and V3, compared to the relatively sensitive, M26 and B118, rootstocks. Thus, it could be inferred that under short-term osmotic stress, the decrease in stomatal conductance in tolerant apple genotypes would have a negative effect on transpiration rate and in turn a positive effect on the RWC. However, it was also apparent that the effect of osmotic stress on carbon assimilation was slightly lower than its effect on transpiration rate, which could explain the increase in WUE under osmotic stress in tolerant rootstocks, B9 and V3. Additionally, the highest content of ABA under stress conditions was shown in B9 followed by V3 and G41 and G935; and the lowest ABA was in M26 and B118. M26 and B118, with low ABA levels, demonstrated the highest stomatal conductance and evaporation among the six rootstocks, suggesting the important role of ABA in improving the tolerance in these rootstocks in the short term.

### 3.2. Changes in ABA Content under Osmotic Stress

ABA is a phytohormone that plays a pivotal role in mitigating the adverse effects of the decreased water potential in plants under osmotic stress by regulating the expression of many osmotic-related genes (ORGs) and regulating several physiological processes [6,17,20,50,52]. In the current study, ABA concentration increased significantly in most apple rootstocks in response to the PEG (30%) treatment (Figure 3). Under stress conditions, ABA accumulates in the roots and translocates to the leaves to induce stomatal closure, hence reducing evaporation from the leaves and maintaining water status [55]. In general, ABA increase is negatively correlated with the changes in g_s_, E and C_i_ and positively correlated with WUE [6,52]. In the present study, the photosynthesis related variables were negatively correlated with ABA changes in apple rootstocks growing under osmotic stress. The decrease in water potential has been associated with the accumulation of leaf ABA, while the increase in WUE was correlated with reduced leaf transpiration and stomatal conductance, pointing to the vital role of ABA [55]. Moreover, several ORGs were regulated under osmotic stress conditions (Figure 4). For instance, the gene expression of *SnRK*, *DREB*, *ERD*, *MYC2* and *NPR1* were up-regulated in response to osmotic stress. The same genes also showed a strong positive correlation with WUE. The differential expression of these genes highlighted their important role in osmotic stress tolerance in apple rootstocks.

The six rootstocks showed an increase in ABA concentration in response to osmotic stress. Further, the rate of increase in ABA levels varied from 20% in B9, which showed the lowest increase rate to 61.5% in M26, which showed the highest increase. Despite the high increase rate of ABA levels in M26, it had the lowest ABA content under stress. These results suggest that the basal, rather than inducible, level of ABA has more impact on osmotic stress tolerance in apple rootstocks. Indeed, Kamboj et al. (1999) [56] found that dwarfing rootstocks generally have more ABA content under normal conditions than vigorous rootstocks. Similarly, we found that B9, V3 and G41, which were previously reported to be more dwarfing than M26, had higher ABA content than M26 under normal conditions, and these rootstocks demonstrated better responses under osmotic stress conditions than M26. In similar studies, dwarfing rootstocks showed more drought tolerance than their vigorous counterparts, e.g., MM111 and M26 [51]. However, Jiménez et al. (2013) [14] found that peach dwarfing rootstock presented a lower tolerance capacity than vigorous rootstock, suggesting that tree size is not always correlated with plant tolerance. Furthermore, ABA-mediated stress tolerance could also be associated with negative effects on tree productivity, especially under long-term drought conditions, where ABA could induce leaf senesce and abscission to reduce water loss through transpiration.

### 3.3. Differential Expression of ORGs in Apple Rootstocks

The transcripts of ORGs in the six apple rootstocks tested in this study showed differential abundance in response to osmotic stress (Figure 4). *SnRK* expression showed higher abundance in V3 and B9 than other rootstocks. SnRK is a positive regulator of the ABA signaling pathway and its activation induces downstream ABRE-binding protein/ABRE-binding (AREB/ABF) transcription factors that in turn activate ABA-responsive genes [57]. The highest expression of *DREB* under stress was noticed in B9 and G41. At the same time, *MYC2* expression had the lowest transcript levels in M26 compared to the other rootstocks under osmotic stress. Given the known role of MYC2 proteins in abiotic stress tolerance [43] and ABA signaling [57], its low expression in M26 could, at least partially, explain the demonstrated sensitivity of M26 to osmotic stress. *ERD* is also reported to be highly regulated under drought and salinity before the onset of ABA, suggesting it could work independently of ABA [32,58,59]. However, our data showed that *ERD* is highly expressed in the rootstocks with high ABA content, e.g., G41, B9 and G935. In fact, *SnRK*, *DREB*, *ERD* and *MYC2* showed higher up-regulation in the rootstocks with higher ABA levels and higher WUE, indicating not only the positive roles of these genes in improving apple rootstock tolerance, but also the crucial role of ABA as a potential master regulator of these proteins under stress. With regard to *WRKY29*, it was down-regulated in all tested apple rootstocks. However, the reduction in *WRKY29* gene expression observed in V3, G41, and B9 was greater than M26, G935 and B118. Some WRKY members function as a positive regulator of stomatal conductance [34]. WRKY29 was also reported to act as a negative regulator of ABA signaling in dormant rice seeds [60]. Therefore, it was not surprising to find that *WRKY29* downregulation in B9 and V3 was correlated with lower stomatal opening and more tolerance to osmotic stress. Indeed, our data showed a strong negative correlation (*r* =−0.82) between the expression of the *WRKY29* gene and WUE. *NPR1* was highly up-regulated in V3 and G41. The importance of NPR1 stems from its role in enhancing antioxidant activity, reducing oxidative stress and inducing plant stress tolerance to abiotic stress [61,62,63]. Vacuolar H^+^ATPASE is another important protein that plays a vital role in ion and proton transport and its role in improving plant tolerance to salt and drought stresses has been reported [47,64]. H^+^ATPASE controls membrane proton pump involved in moderating cell turgor pressure and regulating stomatal movement [64]. Interestingly, our results showed that *ATPASE* displayed an increase in B118 and G935 only. Overall, the differential expression of some ORGs among the tested rootstocks suggested that other pathways, in addition to ABA, might also be involved in the observed response of these rootstocks to osmotic stress.

## 4. Conclusions

Apple rootstocks showed a decrease in RWC and an increase in EL when subjected to osmotic stress. Osmotic stress also resulted in the reduction of photosynthesis-related parameters P_N_, g_s_, E and C_i_. The changes in the physiological parameters in response to osmotic stress were consistently associated with ABA accumulation and molecular changes known to mitigate and confer osmotic stress tolerance. We found that the genetic background could also be related to the different responses and performance of the tested rootstocks. In view of the current results, we concluded that B9, V3, G41 and G935 are more tolerant to osmotic stress than M26 and B118, which was demonstrated by their abilities to maintain up-regulation in some ORGs such as *SnRK*, *DREB*, *ERD*, *MYC2* and *NPR1* that higher water content, membrane integrity and WUE under stress conditions. Additionally, the tolerant rootstocks showed have previously been reported to induce abiotic stress tolerance when overexpressed in transgenic plants. However, it should also be taken into account that rootstocks can modulate the size, yield, hormone content and even gene expression of the scion [65], which could influence the behavior of the grafted plants under abiotic stress conditions [10]. Therefore, future research is required to understand the mechanisms underlying rootstock-scion interactions under stress conditions and how scion’s growth and yield characteristics would modulate the tolerance capacity of the tested rootstocks, e.g., B9 and V3. Our results also suggest that the basal level, rather than the induced accumulation, of ABA could determine the tolerance capacity of apple rootstocks. However, this would require further validation in a much broader study incorporating several apple rootstocks from different genetic backgrounds.

## 5. Materials and Methods

### 5.1. Plant Materials and Samples Collection

Six apple rootstocks, including M26, Vineland 3 (V3), Geneva 935 (G935), Geneva 41 (G41), Budagovsky 9 (B9) and Budagovsky 118 (B118), were evaluated under osmotic stress. One-year-old dormant plants were grown until bud break in the greenhouse of the University of Guelph, Guelph, Ontario, Canada. The greenhouse growth conditions were 300 μmol m^−2^ s^−1^ light intensity, 24 ± 2 °C day and night temperature and diurnal cycle 16 h light/8 h darkness. Two months after bud break, apple rootstocks were characterized under osmotic stress induced by 30% of polyethylene glycol 6000 (PEG) (Sigma-Aldrich, St. Louis, MO, USA), while water was used as a control. All leaf samples were collected three days after the PEG treatment for molecular, biochemical and physiological analyses, immediately frozen in liquid nitrogen, then stored in −80 °C until further use. The third and the fourth leaves from the apical meristem were used to measure photosynthesis related parameters. The fourth leaf was used for ABA quantification and molecular analyses. The fifth and the sixth leaves were used to measure electrolyte leakage and relative water content.

### 5.2. Measurement of Physiological Changes 

Electrolyte leakage (EL) was determined following an earlier method by Bajji et al. (2002) [66]. Total of nine leaf discs (0.5 cm) were collected using a cork borer. The leaf discs were washed in deionized water and kept on the shaker at 100 rpm at room temperature for 24 h. Initial electrical conductivity (IEC) was measured after 24 h using an electrolyte conductivity meter (Eutech Instruments, PCSTester 35 (ThermoScientific, Vernon Hills, IL, USA)). Later, the samples were autoclaved at 121 °C for 20 min to destroy all membranes and release all electrolytes. The final electrical conductivity (FEC) was measured when the samples reached room temperature. Electrolyte leakage was calculated by the formula: EL = (IEC/FEC) × 100. The relative water content (RWC) was recorded according to Aneja et al. (2015) [67]. Briefly, the fresh weight (FW) of leaf segments was measured immediately after separation from the plant. The leaves were then immersed in distilled water at room temperature for 24 h, and the turgid weight (TW) was recorded. The leaves were dried in the oven for 24 h at 70 °C; finally, the dry weight (DW) was recorded. The RWC was calculated using the formula: RWC (%) = (FW − DW)/(TW − DW) × 100.

Net photosynthesis rate (P_N_), stomatal conductance (g_s_), transpiration rate (E) and inner CO_2_ (C_i_) were measured three days after PEG treatment using LICOR photosynthesis system LI-6400 XT (Li-Cor, Inc., Lincoln, NE, USA). In addition, water use efficiency (WUE) was calculated by dividing the photosynthesis rate by the transpiration rate (WUE = P_N_ /E). Measurements were conducted between 10:00 am and 12:00 pm (GMT) in three plants for each treatment. Parameters were measured at a light intensity of 1000 µmol m^−2^ s^−1^ provided by an external light source, 400 µmol CO_2_ mol^−1^ and leaf temperature of 25 °C.

### 5.3. Extraction and Analysis of ABA

The fourth leaf from the apical meristem was collected three days after the PEG treatment, ground in liquid nitrogen to fine powder, then freeze-dried. Around 100 mg of freeze-dried tissues were treated with 1 ml extraction buffer consisted of 50% methanol (MS Grade, Fisher Scientific, Mississauga, ON, Canada; MeOH) and 4% acetic acid (Fisher Scientific, Mississauga, ON, Canada) in Milli-Q water. The samples were then sonicated on ice for 30 min and centrifuged for 2 min at 13,000 rpm. After that, the supernatant was removed to a new tube and diluted 5× in 10 mM ammonium acetate (Fisher Chemical, Fair Lawn, NJ, USA), pH 9 adjusted with ammonium hydroxide (Sigma Aldrich, Mississauga, ON, Canada). Then 500 µL of samples were filtered-centrifuged using 0.45 µm Millipore centrifuge filter at 13,000 rpm for 1 min. Finally, the supernatant was used for quantification using ultra-performance liquid chromatography (UPLC)—mass spectrometry. As described by Erland et al., 2017 [68], 3 µL of sample were injected onto a Waters Acquity BEH Column (2.1 × 50 mm, i.d. 2.1 mm, 1.7 µm) on a Waters Acquity Classic UPLC system with detection using an Aquity QD a single quadrupole mass spectrometer (MS) controlled by Empower 3 (Waters, Canada). Samples were run on a gradient with A-10 mM ammonium acetate pH 9, adjusted with ammonium hydroxide; B—100% MeOH with initial conditions of 95% A 5% B increased to 5% A 95% B over 4.5 min using Empower curve of 8. Column temperature was 40 °C and flow rate was 0.5 mL/min. Capillary voltage was 0.8 kV, and probe temperature was 500 °C with a gain of five. ABA was monitored in single ion recording mode and quantified ng/g dry weight (DW) using a standard curve.

### 5.4. RNA Extraction and Gene Expression Analyses

Tissue samples were ground to a fine powder in the presence of liquid nitrogen, after which total RNA was extracted using CTAB as previously described by Gasic et al. (2004) [69]. cDNA synthesis was carried out using 2500 ng of purified and DNase-treated RNA in a 20 µL reverse transcription reaction mixture using a High-Capacity cDNA Reverse Transcription Kit (Thermo Fisher Scientific, V.A. Graiciuno, Vilnius, Lithuania) following the manufacturer’s instructions. The cDNA was diluted 1/10 with ultrapure water. The expression analysis of the selected osmotic-related genes (ORGs) was quantified for three biological and three technical replicates for each sample using a CFX Connect Real-Time System (Bio-Rad, Hercules, CA, USA), the EvaGreen Supermix (Bio-Rad, Hercules, CA, USA), and gene-specific primers (Table 1). The expression of each gene was normalized to that of elongation factor 1A (*EF1A*) and was calculated relative to M26 under control (unstressed) condition. Relative normalized expression was calculated using Bio-Rad CFX Manager 3.1 software (Bio-Rad, Hercules, CA, USA) and according to the 2^−∆∆CT^ method [70].

### 5.5. Statistical Analysis

The experiment was arranged as a two-factorial in a randomized complete block design with three blocks and three plants in each block. The results were confirmed by repeating the experiment twice. Mixed models including rootstock and osmotic stress as fixed factors and block as a random factor were performed to distinguish treatment effects for all tested parameters. Statistical analyses were performed using SAS Institute Inc., Cary, NC, USA. Means were subjected to analysis of variance (ANOVA) at *p* < 0.05, and the significant differences were compared by Tukey-Kramer multiple means comparison. The log model was used to transfer the data to follow normal distribution when required. Pearson correlation test was performed using GraphPad Prism v9 to correlate the physiological, molecular changes and ABA content in apple leaves.

## Figures and Tables

**Table 1:** List of the used primers of osmotic responsive genes in apple (*Mulas domestica*).

Accession #	Gene Name	Forward Primer	Reverse Primer
DQ341381.1	*EF1A*	ATTCAAGTATGCCTGGGTGC	CAGTCAGCCTGTGATGTTCC
NM_001294018.1	*DREB*	GCAATTACAGGGGAGTGCG	ATAGGCAAGGGCAGCATCA
JX569851.1	*SnRK*	AGCCAAAATTCCTCCTCCA	TTCTTCCTCCTCGCCTTCT
XM_029092593.1	*ERD*	TTTATCCCTGCGGCTCTCC	CTGAGCCAGTAGTCGTGGT
EF128033.1	*ATPASE*	TTGAGGATCCAGCTGAAGG	CAAGAGCACGGAAACCACT
XM_008377742.2	*WRKY29*	AGCTGTGGTAAGAGGGTGC	GGCTTCAAAGGCCTGAGGA
NM_001328944.1	*MYC2*	GCAACGAGGAGGGGATATT	GTCCGAGTGGTCTGAATCG
>DQ074459.1	*MYB2*	AGCCACCGAACAGCCTAAT	TGGAATCGGCCTTGGGAAT
XM_008392806.2	*NPR1*	CGTGGTGAGGTCTAATGGTG	TTGGGTGCCAATGTTCTCTC

## Data Availability

All data generated or analyzed during this study are included.

## Abbreviations

Osmotic stress (OS), osmotic-responsive genes (ORGs), polyethylene glycol (PEG), abscisic acid (ABA), electrolyte leakage (EL), relative water content (RWC), net photosynthesis rate (P_N_), stomatal conductance (g_s_), transpiration rate (E) and inner CO_2_ (C_i_), water use efficiency (WUE).
